# MYC’xing it up: zebrafish B ALL models provide insight into MYC-driven disease and relapse

**DOI:** 10.18632/oncotarget.27644

**Published:** 2020-06-23

**Authors:** Diana Sá da Bandeira, Wilson K. Clements

**Keywords:** zebrafish, leukemia, B ALL, MYC, relapse

***News on:*** *Zebrafish B cell acute lymphoblastic leukemia: new findings in an old model by Park et al. Oncotarget. 2020; 11:1292–1305. https://doi.org/10.18632/oncotarget.27555*

In this issue of *Oncotarget*, Park *et al*. present recent studies of zebrafish models of B lineage acute lymphoblastic leukemia (B ALL) driven by human and mouse *c-MYC* oncogenes. The comparative gene networks driven by the two MYC isoforms are surprisingly distinct. They further show that B ALL driven by human *c-MYC* (h*MYC*) is responsive to induction of remission using typical frontline therapies, including radiation and dexamethasone, and susceptible to subsequent relapse. These studies highlight an unusual animal model of B ALL with potential for investigation of the genetic and protein modifiers of MYC-driven B ALL and relapse disease.

Genetically engineered models of malignancy are a mainstay of cancer research. They allow investigation of key features of disease including how defined genetic lesions contribute to transformation, disease etiology and progression, tumor biology, and drug responsiveness. Two features of disease that have been difficult to address using genetic models are how distinct disease entities arise from apparently similar genetic lesions, and the biological basis for drug responsiveness and relapse. Although the predominant genetic cancer model has historically been mouse, zebrafish models of malignancy have gained popularity over the last fifteen years, owing to their high experimental tractability, receptivity to direct visualization of tumor biology in conjunction with availability of diverse transgenic fluorescent reporter lines, and importantly, the high level of conservation with humans in genetic regulation of normal and malignant behavior. The comparatively large number of zebrafish that can be produced via single matings, and their relative cost- and space-effectiveness to raise and maintain, further recommend them as a vertebrate model uniquely suitable for studies that would benefit from availability of large numbers of individuals, notably pharmacological studies where it would be beneficial to have many comparison strata, and relapse studies where initial disease and treatment may eliminate a significant number of study individuals.

Acute lymphoblastic leukemia (ALL) is the most common childhood cancer and the second-most common acute leukemia in adults. ALL frequently displays T or B lineage identity, and less commonly, mixed lineage [1]. Although overall cure rates for pediatric ALL now exceed 90% in most developed countries [1], specific subtypes, adult disease, and relapse cases have significantly worse prognosis. As current therapies often lead to debilitating long-term side effects and some patients are unresponsive to treatment, or relapse with significantly worse prognosis, there is an urgent need to develop targeted therapies and improved relapse therapies. Animal models suitable for understanding the biological basis of initial response and relapse potential are essential for these goals. Although there are now a number of animal models of hematologic malignancy, B lineage-specific models have been comparatively rare.

**Figure 1:**
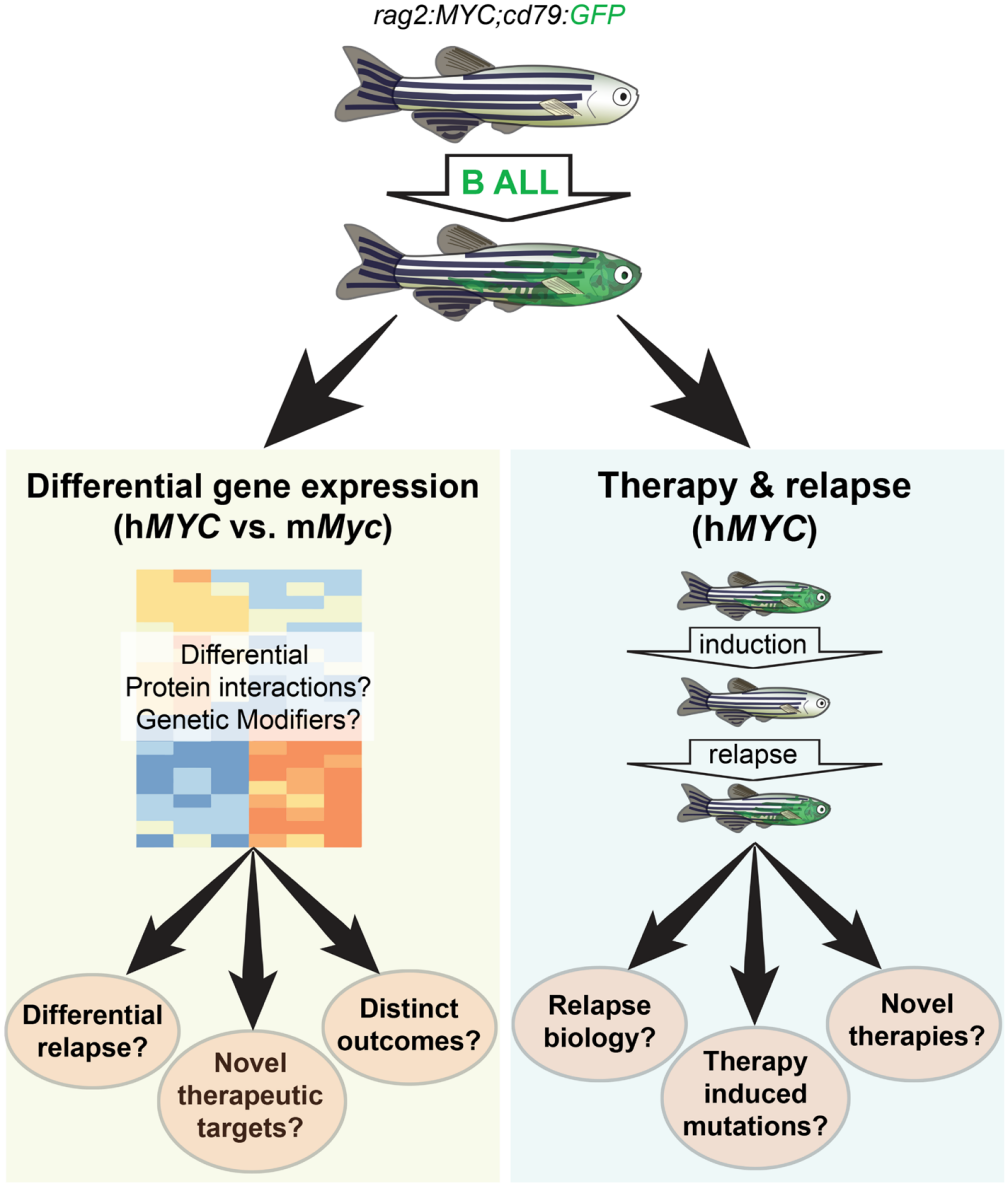
Uses of a zebrafish model of B lineage acute lymphoblastic leukemia (B ALL). Recent studies have demonstrated that transgenic zebrafish carrying the *c-MYC* oncogene under the control of regulatory elements from the *rag2* gene (*rag2:MYC*) develop B lineage leukemia in addition, and sometimes in parallel to T lineage leukemia. The B lineage reporter *cd79:GFP* transgene facilitates green fluorescence-based identification of tumor-bearing individuals. Comparative genomic profiling of human vs. mouse tumors (left) reveals differences between tumors resulting from human and mouse *MYC* isoforms, which might be due to differential protein isoforms, genetic modifier loci, or both. Comparison of the two models might provide insight into how distinct disease entities arise from superficially similar genetic lesions, provide clues to druggable genetic targets mediating MYC effects, or provide clues to relapse potential. Treatment of zebrafish carrying h*MYC* with dexamethasone or radiation results in induction of remission and relatively speedy relapse, providing a model for understanding relapse biology, identifying treatment induced mutations, and possible discovery of novel therapeutic strategies for relapse disease.

The first zebrafish malignancy model of any kind (Figure 1) was created by placing a mouse *Myc* oncogene under the control of regulatory elements from the endogenous zebrafish *rag2* promoter [2]. The transcription factor MYC is deregulated and overexpressed in the majority of human malignancies including B lineage malignancies [3]. In early studies describing the c-MYC-driven model of lymphoid malignancy, investigators described a robust, highly penetrant, and aggressive T lineage malignancy, both in the original transgenic line using mouse *c-Myc* (m*Myc*) and in the second later h*MYC* line. Surprisingly, more than a decade later, two groups independently verified that these models also developed previously unappreciated and less frequent B lineage malignancies, as well as occasionally mixed lineage leukemias [4, 5].

In this issue of *Oncotarget*, Park *et al*. report B ALL comparative tumor transcriptional profiling results for zebrafish h*MYC* and m*Myc*, as well as drug responsiveness and relapse potential in the zebrafish h*MYC* model (Figure 1). Interestingly, the mouse- and human-driven diseases, though superficially similar, appear to instruct expression of surprisingly distinct gene networks [6]. In most malignancies where it is expressed, MYC requires the presence of additional cooperating oncogenes or genetic lesions, emphasizing the importance of understanding context. In the current study, Park *et al*. found that despite overwhelming similarity of the human and mouse MYC proteins, distinct genetic networks are expressed downstream, exhibiting differential expression of cell cycle regulators or *fos* and *jun* family members. It is not yet clear whether these differences are the consequence of intrinsic differences between the human and mouse MYC protein isoforms, strain-specific genetic modifiers that might be relevant to disease etiology, or both. Defining the basis for these differences in the future may be informative for understanding potential differences in distinct MYC-driven malignancies. Moreover, MYC itself has generally been considered “undruggable” [7], highlighting the importance of defining downstream genetic networks to identify potential nodes receptive to therapeutic intervention.

The authors also demonstrate that the h*MYC* B ALL zebrafish model is responsive to key frontline human treatments, including dexamethasone and radiotherapy, and are susceptible to highly penetrant and aggressive relapse. These findings elevate the potential clinical relevance, demonstrating that the zebrafish disease shares treatment sensitivity with human B ALL, therefore suggesting that it might represent a good model for discovery of new interventions, and that treatment-induced genetic alterations might be conserved. Developing robust models of relapse has been a major challenge for understanding disease progression, clonal evolution, and drug responsiveness.

Recent studies have indicated that in pediatric ALL, a primary mechanism of relapse disease is therapy-induced mutation, rather than *de novo* resistance or escape of rare, therapy-resistant clones [8]. In the future, it will be interesting to understand what additional therapeutic modalities are effective in zebrafish B ALL models and compare the pre- and post-therapy gene expression in these models to better understand whether they exhibit a similar set of acquired mutations to those found in humans. For example, do relapsed individuals exhibit mutations in glucocorticoid transcriptional response factors, as might be expected for individuals relapsing from dexamethasone-induced remission? If so, perhaps zebrafish may eventually become a model for discovery of key MYC networks driving differential disease progression and relapse, investigation of how new treatments can be combined with existing modalities, and even pharmacological discovery for targeted therapies and relapse disease.
